# Design on the Bowden Cable-Driven Upper Limb Soft Exoskeleton

**DOI:** 10.1155/2018/1925694

**Published:** 2018-07-17

**Authors:** Wei Wei, Zhicheng Qu, Wei Wang, Pengcheng Zhang, Fuchun Hao

**Affiliations:** ^1^College of Physics, Optoeletronics and Energy and Collaborative Innovation, Soochow University, Suzhou 215000, China; ^2^College of Rehabilitation Therapeutic Specialty, Tianjin Medical College, Tianjin 300000, China

## Abstract

To assist hemiplegic patients with the activities of daily life, many upper limb soft exoskeletons have been developed. In this paper, we propose the structure of upper limb soft exoskeleton for rehabilitation training based on human biomechanics. The soft driving structure based on Bowden cable is devised. Man-machine interaction force must be considered because it can damage on the joint and lead to arm discomfort. We focus on structural optimization to minimize man-machine interaction force. Human arm model is established to perform motion simulation in ADAMS. To summarize optimality conditions, the movements of elbow are simulated in ADAMS when the number and location of force bearing points are changed. This paper describes the movement of the shoulder skeletal system through a mathematical model based on the Bowden cable transmission and utilizes man-machine contact force sensor to detect human interaction forces for analysis of experimental data. The experimental results show that man-machine interaction force can be reduced when the number of bearing force points is increased and bearing force point is away from the elbow.

## 1. Introduction

In recent years, many wearable exoskeletons were developed rapidly in the field of assistance and rehabilitation [1]. Exoskeletons have been applied to provide biological joint assistive torques to strengthen the sport ability of human body or assist the person with disability [2]. These wearable exoskeletons are usually made up of rigid links that run in parallel with the biological limb [3]. Rigid structure is substituted by soft structure to overcome discomfort resulting in rigid exoskeleton. The pneumatic artificial muscle actuator is used to replace the traditional motor and hydraulic drive [4]. Even if rigid structures are made of aluminum and clad metal, the entire weight of exoskeleton with pneumatic artificial muscles can be reduced greatly. However, the discomfort and joint injury aroused by mechanical impedance and kinematic constraints have not been well solved [5].

In order to eliminate these limitations, concept of soft exoskeleton was put forward. Firstly, a novel soft cable-driven exosuit which can apply forces to the body to assist walking was proposed [6]. The binding structure consisted of textile fabric without rigid connections and hinges. Human body is connected to soft exoskeleton through the quasioptimal compliant interface. To solve the limitations of stretch elongation for the pneumatic actuator, a new type of actuator based on Bowden cable for soft wearable exoskeletons was put forward [7]. This actuation structure allows the motor to be placed on better-loaded parts away from the motion joint. The extremity of Bowden cable sheath is connected to pulley cover, another extremity is connected to the anchor point. Soft exoskeleton transmits driving force through inner Bowden cable, and the pulling force of Bowden cable is converted to joint torque [8]. The force transfer effect of the Bowden cable is good. Hence, the actuation structure based on Bowden cable transmission is becoming the first choice of design.

At present, the number of stroke patients in China is over 2 million each year. About 75% of stroke survivors have different degrees of disability. Apoplectic hemiplegia is one of the common sequelae. Targeted treatments for apoplectic hemiplegic patients include the functional recovery of shoulder, elbow, hip, and knee [9]. While the stroke hemiplegia patient with upper limb soft exoskeleton performs rehabilitation training, man-machine interaction force can inflict damage on human body. The chief solution is to reduce the man-machine interaction force in the supporting process by optimizing control strategies. Hierarchical cascade controller was proposed to improve control strategy for upper limb soft exoskeleton. The high-level controller performs assistance level estimation, midlevel controller performs adaptive gap compensation, and low-level controller performs adaptive friction compensation and position control [10]. In this paper, the structural optimization is mainly studied to reduce man-machine interaction force. The selection of the fixed anchor position between the Bowden cable and the surface textiles of the human body has a great influence on the comfort and compliance. The number of Bowden cables also has an impact on man-machine interaction force.

This paper primarily introduces three parts. Firstly, on the basis of the existing research on soft exoskeleton, a structure of upper extremity soft exoskeleton for the rehabilitation training is proposed. Then, the influence factors of human-machine interaction force are discussed and some methods of structural optimization are put forward. Finally, the subject with upper limb soft exoskeleton performs flexion movements of elbow to verify the conclusions presented in simulation experiments. The man-machine interaction force can be decreased when force bearing points are added and away from the center of elbow in a certain range.

## 2. Structure Design

### 2.1. Design Overview

**Figure 1:**
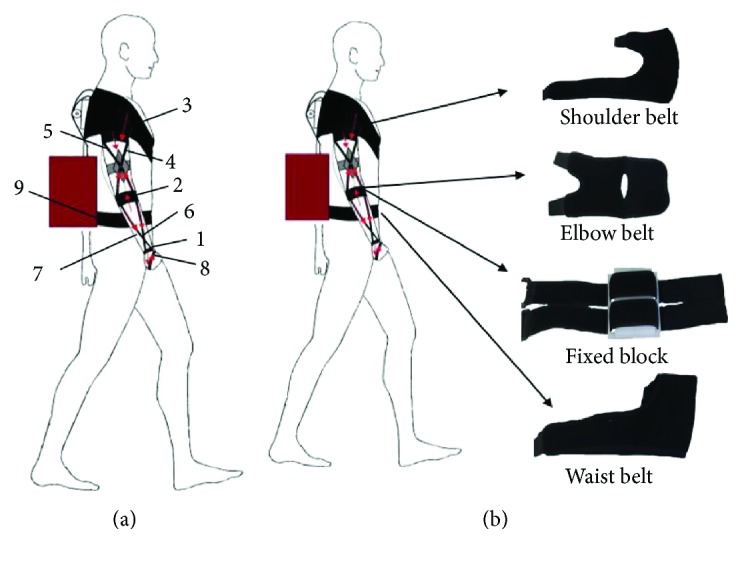
Overall structure of upper limb soft exoskeleton.

Based on the analysis of the human upper limb biomechanics, textile materials constitute the basic structure of soft exoskeleton [11]. Force transfer of soft exoskeleton is shown in Figure 1. The numbers mentioned in this article are labels in the figure. The upper limb soft exoskeleton attaches around the shoulder (3), below the elbow (2) and around the waist (9). Force is transmitted through the straps (4–8). The extremity of Bowden cable sheath is attached to a fixed point of the webbing strap (3) around the shoulder. The inner Bowden cable extends from the extremity of Bowden cable sheath down to the fixed point of the webbing strap (2) below the elbow. When extension movements of elbow are performed with assistance delivered by soft exoskeleton, the distance between fixed points on the straps (2) and (3) is pulled closer. Strap (2) transfers the downward force to strap (1) when flexion movements of elbow are performed. At the same time, strap (2) transmits the upward force to strap (3).

The upper limb soft exoskeleton produces torque at the elbow through Bowden cable transmission system. The operation principle of muscle tendon can be simulated by Bowden cable transmission system. Further, man-machine coupling coefficient of flexible structure is high. Part of the driving force is generated passively by the human body, and the other is provided by the exoskeleton. Referring to Figure 1, when flexion movements of elbow are performed, the strap (2) gradually tightens. The strap (2) generates impedance torque while the elbow is flexed to the maximum extent. When extension movements of elbow are performed, the strap (2) releases the stored energy and assists the elbow movement.

The webbing belts are mode of pump-effect materials to enhance breathability and comfort. The waist belt is constructed to be a single piece to be secured in the front with Velcro. The Velcro on the waist belt can fit waist sizes within a 10 cm range. It is more convenient to don and stretches less due to Velcro than buckle form. The shoulder belt is made into “I”-shape and has two independently adjustable Velcro-covered tabs along its height, enabling it to be secured to the subject's shoulder in a comfortable manner. A layer of fabric sewn on the shoulder belt is utilized to fix the Bowden cable sheath, and the interior is filled with sponge to reduce pressure.

The elbow belt is constructed to be a single piece and has two independently adjustable Velcro at the end of belt to fit elbow sizes within a 8 cm range. The belt which is utilized for force transmission between shoulder belt and elbow belt is sewn up, and the width of belt is 3 cm.

There are two fixed blocks made of ABS plastic to fix the Bowden cable between the upper arm and forearm. Each fixed block has two independently adjustable Velcro, and the Velcro can fit upper arm and forearm sizes within a 5 cm range. The weight of the entire upper limb soft exoskeleton structure is less than 800 g. Suitable length of Bowden cable is selected to prevent excessive bending and acts as a mechanical limit to protect the subject.

### 2.2. Design Principles

The role of upper limb soft exoskeleton is to assist hemiplegic stroke patients for rehabilitation training. In order to avoid the damage resulting in displacement of webbing straps, overall stiffness should be improved. It is less possible to lead to damage when the overall stiffness is higher. Therefore, a few principles should be followed when designing the upper limb soft exoskeleton for patients. Certain areas of the human body are more load bearing, and this feature needs to be fully taken into account. These areas are defined as key anchors such as shoulders, buttocks, and soles. These typical areas are mainly composed of bones which can withstand normal or near-normal reaction forces. The primary structure of soft exoskeleton is located on the shoulder. During the driving stage, the skin has less displacement relative to the bone. Webbing strap (3) provides the humerus upward tension to prevent downward and lateral movement of the humerus. So, the shoulder with soft exoskeleton can remain relative stable.

Most of the load is transferred to the pelvis to ensure relative movement of the bone and skin as normal as possible when the body is loaded. Shearing forces can result in damage to the human body if it exceeds the friction force of the skin. It is impossible to completely avoid the shearing force between the soft exoskeleton and human body. A little displacement can occur in skin and bone under low shearing force. For example, when flexion movements of the elbow are performed, a few displacement at the elbow cannot give rise to damage to human body. In the binding structure of soft exoskeleton, the displacement under the normal force hardly results in harm to the human body.

The wide webbing strap can be utilized to reduce the pressure between soft exoskeleton and human body. The body can withstand some degree of pressure before discomfort. The estimated maximum comfort pressure is usually around 0.5 N/cm. When contact area of webbing strap is increased, the displacement of webbing strap can be reduced to increase the overall stiffness. In addition to the increase in the width of webbing strap, pressure can be reduced when the tension on the webbing straps is balanced. If the patient with soft exoskeleton carries out rehabilitation training, the comfort of the patients can be improved by adjusting the balance tension of the webbing straps (4–7). Due to the particularity of hemiplegia stroke patients, the Bowden cable sheath is connected to the fixed position through a pulley mounted on the back to avoid harm to body.

### 2.3. Structure of Actuator

Considering that the upper limb soft exoskeleton is used for rehabilitation training, the maximum torque generated by DC motor must help the patients to lift the forearm passively. The maximum torque delivered by actuation system can be defined as follows:

(1) $$\begin{array}{c} \tau_{m}=W\times L, \end{array}$$

where *τ*_m_ is maximum torque provided by DC motor, *W* is the weight of forearm, and *L* is the distance from the centroid of the forearm to the center of elbow [12].

**Figure 2:**
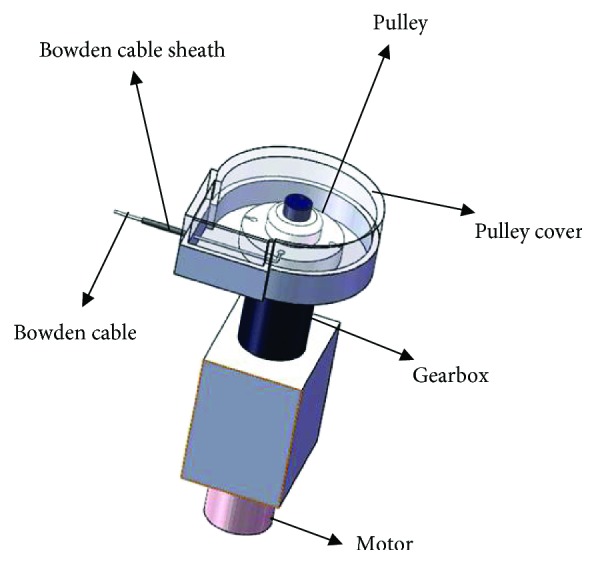
A 3-D model of the actuator system.

As shown in Figure 2, it is an actuation system of upper limb soft exoskeleton. The actuation system is composed of a Maxon EC 45 251601 Flat brushless DC motor and a 5 : 1 planetary gearbox connected to the DC motor. Bowden cable sheath is wrapped outside the Bowden cable to prevent damage resulting in Bowden cable during transmission. The extremity of the Bowden cable sheath is connected to the outer frame of the pulley cover. Another extremity is connected to the bottom of the rear arm by means of a pulley mounted on the back. The extremity of the Bowden cable is connected to the pulley, and the other is connected to the fixed anchor of the forearm [13]. The Bowden cable is pulled tight, and torque is generated at the elbow during driving stage. This actuator primarily delivers torque generated by the motor to the soft exoskeleton through the Bowden cable. Hence, the actuator can be installed in a position away from the elbow. The patient with upper limb soft exoskeleton is subjected to a smaller load and improves comfort during rehabilitation training.

### 2.4. Selection and Calibration of Sensors

The flex sensor 4.5^″^ (SparkFun, USA) acts like a variable resistor whose output voltage is changed by flexing it. We utilize a simple magnified voltage divider to convert a variation in resistance into a variation in voltage. The relationship between the output voltage and the bending angle is assumed to be linear [14]. The flex sensor needs to be calibrated to obtain the mapped relationship between the output voltage and the bending angle before experiment to render the measurement. A film pressure sensor DF9-40@10 kg (LEANSTAR, China) is a sensor for measuring the degree of pressure. When the external pressure acts on the film pressure sensor, the resistance value of the sensor is changed. The change in external pressure is converted into the change in voltage by a voltage divider. According to the manual of data, the film pressure sensor is calibrated to establish the linear mapped relationship between the output voltage and the pressure value. Due to the interference of environment and other factors, the Butterworth filter is utilized to filter the noise when the sensor is connected to the circuit.

## 3. Structural Optimization

### 3.1. Dynamic Analysis of Upper Limb Soft Exoskeleton

Due to the different motion abilities of patients, the torque provided by soft exoskeleton changes with the patients' condition. The relationship among force on Bowden cable, body torque, and the motion angle is obtained by establishing a mathematical model based on the Bowden cable transmission [15].

**Figure 3:**
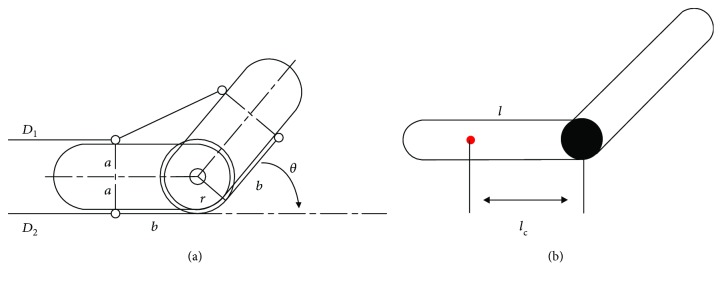
(a) The soft exoskeleton with Bowden-cable transmission worn by the subject. (b) Human arm model.

The human arm model based on the Bowden cable transmission is shown in Figure 3(a). The objective function *D*_1_(*θ*) of the flexible Bowden cable is defined as

(2) $$\begin{array}{c} D_{1}\left(\theta \right)=2\sqrt{a^{2}+b^{2}}\cos\left(\tan^{-1}\left(\frac{a}{b}\right)+\frac{\theta }{2}\right)-2b. \end{array}$$

The objective function *D*_2_(*θ*) of the extensor Bowden cable is defined as

(3) $$\begin{array}{c} D_{2}\left(\theta \right)=r\theta , \end{array}$$

where *a* is half of the width of arm, *b* is the distance from the center of the elbow to the fixed point, *r* is the radius of the elbow, and *θ* is the angle of the flexion/extension movements. Matrix **J** is defined as

(4) $$\begin{array}{c} J\left(\theta \right)=\frac{\partial D^{T}}{\partial \theta }\left(\theta \right),\\ D={\left[\begin{array}{cc} D_{1}\left(\theta \right) & D_{2}\left(\theta \right) \end{array}\right]}^{T}. \end{array}$$

Assistance torque of Bowden cable is defined as

(5) $$\begin{array}{c} \tau =J\left(\theta \right)f,\\ f={\left[\begin{array}{cc} f_{1} & f_{2} \end{array}\right]}^{T}, \end{array}$$

where *f* is the tension of Bowden cable detected by the load cell. The human arm dynamics can be established by the Lagrange formula [16]. The kinetic energy and potential energy of the arm are defined as follows:

(6) $$\begin{array}{c} T=\frac{1}{3}{ml}^{2}{\dot{\phi }}_{b}^{2},\\ V={mgl}_{c}-{mgl}_{c}\cos\phi_{b}, \end{array}$$

where *m* is the quality of the human arm, *l* is the length of human arm, and *l*_c_ is the distance from the center of gravity of the forearm to the center of the elbow. By defining the Lagrange function *L* = *T* − *V* and Lagrange formula, a dynamic model of the human arm is established as

(7) $$\begin{array}{c} \tau_{h}=\tau_{t}-\tau_{a}=\frac{2}{3}{ml}^{2}{\ddot{\phi }}_{b}+{mgl}_{c}\sin\phi_{b}-J\left(\theta \right)\left[\begin{array}{cc} f_{1} & f_{2} \end{array}\right], \end{array}$$

where *τ*_t_ is the total moment of the human arm and exoskeleton, *τ*_a_ is the estimated moment of exoskeleton, and *τ*_h_ is the moment of the human arm.

The upper limb soft exoskeleton system detects man-machine interaction force in real time through the flex sensor and feeds back to control system, so that the exoskeleton system can work stably and accurately. The soft binding structure enables high degree of man-machine coupling and maintains the stability of exoskeleton system to a certain extent without professional protect.

### 3.2. Motion Simulation

The impact of man-machine interaction force on wearers needs to be taken into account because the subject of upper limb soft exoskeleton is hemiplegic patients. Considering the interaction forces between the upper limb soft exoskeleton and human being, two factors need to be considered: pressure distribution and pressure size. The former reflects the comfort of the wearer, and the latter is related to the safety of the patient [17]. In general, there are two main ways to reduce the man-machine interaction force. The method is that greater pressure is exerted on a place with greater resistance. Another method is that the pressure is dispersed in as large as a range as possible to reduce the pressure on the skin. Some parts of the body are not suitable for man-machine interaction force. The following points should be paid attention to. In order to ensure the freedom of movement for joint, it is not possible to choose the contact point around the active joint. Nerve intensive areas should be evaded to avoid unnecessary damage [18]. Due to the diversity of patients' body and athletic ability, we need to find a universal optimization structure which can improve the comfort of patients. The upper limb soft exoskeleton transfers the driving force through the Bowden cable connected to the webbing strap of body. On the one hand, we should take into account the influence of number of Bowden cables on man-machine interaction force; on the other hand, we should consider the influence of fixed position between the Bowden cable and the webbing strap on man-machine interaction force.

**Figure 4:**
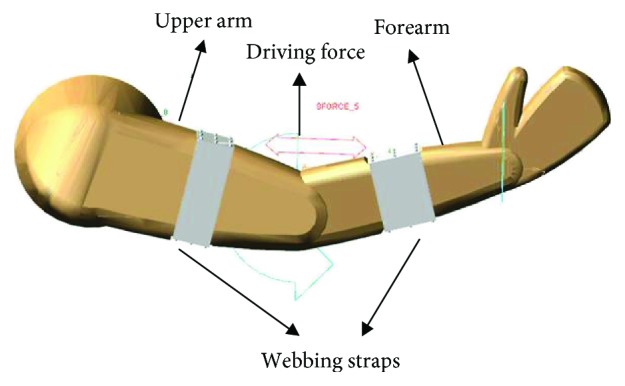
Human arm model built in ADAMS.

As is shown in Figure 4, a human arm model with soft exoskeleton is established in ADAMS. The rotation angle of the human elbow model is 60 degrees [19]. The flexion movements of elbow with assistance provided by soft exoskeleton are simulated under different conditions to study the variation curve of man-machine interaction force.

**Figure 5:**
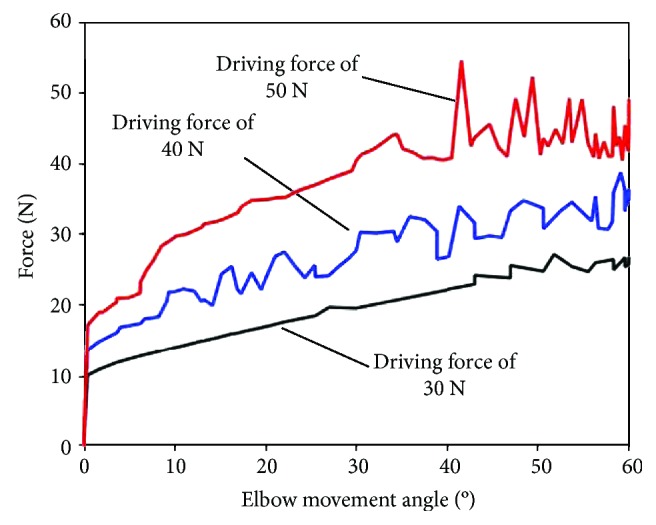
Man-machine interaction force at different tensions.

Firstly, the influence of the size of driving force on the man-machine interaction force is analyzed. Results of simulation experiments comparing the man-machine interaction force with different driving forces are depicted in Figure 5, showing that the man-machine interaction force is enhanced with the increase of driving force. The spread of pressure in a larger range is often proved to be a good way to eliminate the pain and the resulting damage [20]. The transmission of man-machine interaction force between human and soft exoskeleton is affected by the soft tissues of the human body.

The relationship between man-machine interaction force and the deformation of human skin soft tissue is established. Soft tissue models are usually divided into uniaxial and multiaxis models, and each model can be divided into nonlinear elastic and viscoelastic elements. In the nonlinear model, the relationships between stress *T* and strain *ε* are expressed as

(8) $$\begin{array}{c} \varepsilon^{2}={aT}^{2}+bT,\\ T=\frac{F}{S_{0}},\\ \varepsilon =\frac{\left(L-L_{0}\right)}{L_{0}}. \end{array}$$

In elastic fibers, the relationship between stress and strain is expressed as *ε*^2^ = *a*(1 − *e*^*bT*^). The elastic model of the skin is expressed as *T* = *aε*^*b*^. The linear viscoelastic characteristics of the soft tissue are described by the Maxwell element and the Voight element. The equation of Maxwell element is expressed as follows:

(9) $$\begin{array}{c} k\left(t\right)=k_{i}e^{-\left(t/\lambda_{i}\right)},\\ \lambda_{i}=\frac{B_{i}}{k_{i}}, \end{array}$$

where *k* is the stiffness coefficient and *B* is the damping coefficient. The equation of Voight element is expressed as follows:

(10) $$\begin{array}{c} J\left(t\right)=J_{i}e^{-\left(t/\lambda_{i}\right)},\\ J_{i}=\frac{1}{k_{i}}. \end{array}$$

**Figure 6:**
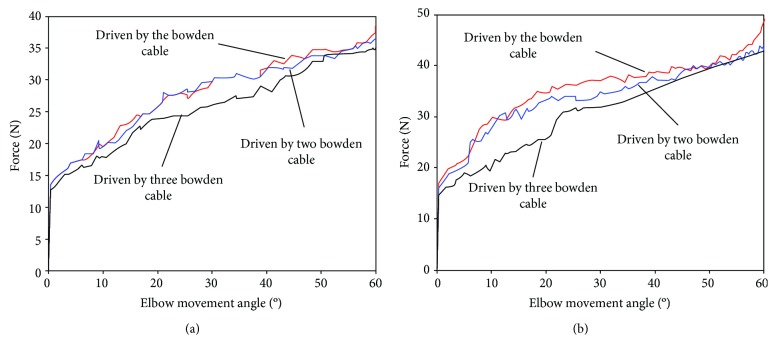
Man-machine interaction force at different numbers of Bowden cables. (a) The flexion movement of elbow under the driving force of 40 N. (b) The flexion movement of elbow under the driving force of 50 N.

The man-machine interaction force is studied when the number of Bowden cables connected to the fixed point on the webbing strap of forearm is changed. According to the spatial integration theory, as the pressure contact area increases, the number of surface pressure sensors also increases and human comfort will deteriorate. Therefore, the maximum number of Bowden cables is set to three. Results of simulation experiments comparing the man-machine interaction force with different numbers of Bowden cables are depicted in Figure 6, showing that interaction force is decreased with the increase of the number of Bowden cables. As the number of Bowden cables is increased, force bearing points on webbing strap are added and force exerted by a single connection point is decreased.

**Figure 7:**
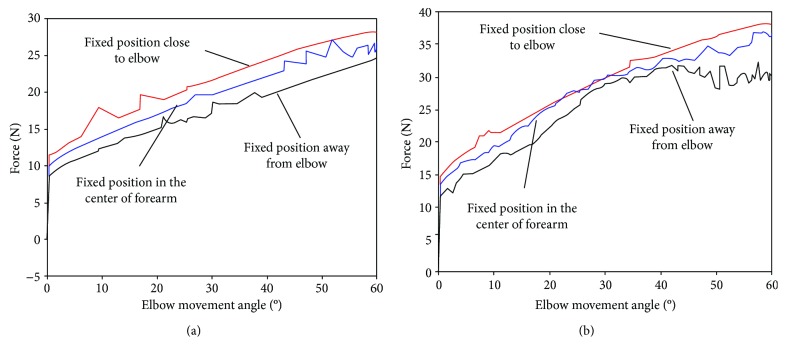
Man-machine interaction force at different force bearing points. (a) The flexion movement of elbow under the driving force of 30 N. (b) The flexion movement of elbow under the driving force of 40 N.

The man-machine interaction force is studied based on different force bearing points on the webbing strap of forearm. The center of the forearm is defined as the middle position. The position which extends three meters from the middle position to the wrist is defined as the position away from the elbow. The position which extends in the opposite direction is defined as the position close to the elbow. Results of simulation experiments comparing the man-machine interaction force with different force bearing points are depicted in Figure 7. Based on webbing strap of forearm built in ADAMS, the farther the force bearing point is from the center of elbow, the smaller the man-machine interaction force is.

From the simulation results, it is concluded that the man-machine interaction force between the human body and the exoskeleton can be reduced by changing the number of Bowden cables and connection point on the webbing strap of forearm.

## 4. Experimental Evaluation

### 4.1. Experimental Scheme

**Figure 8:**
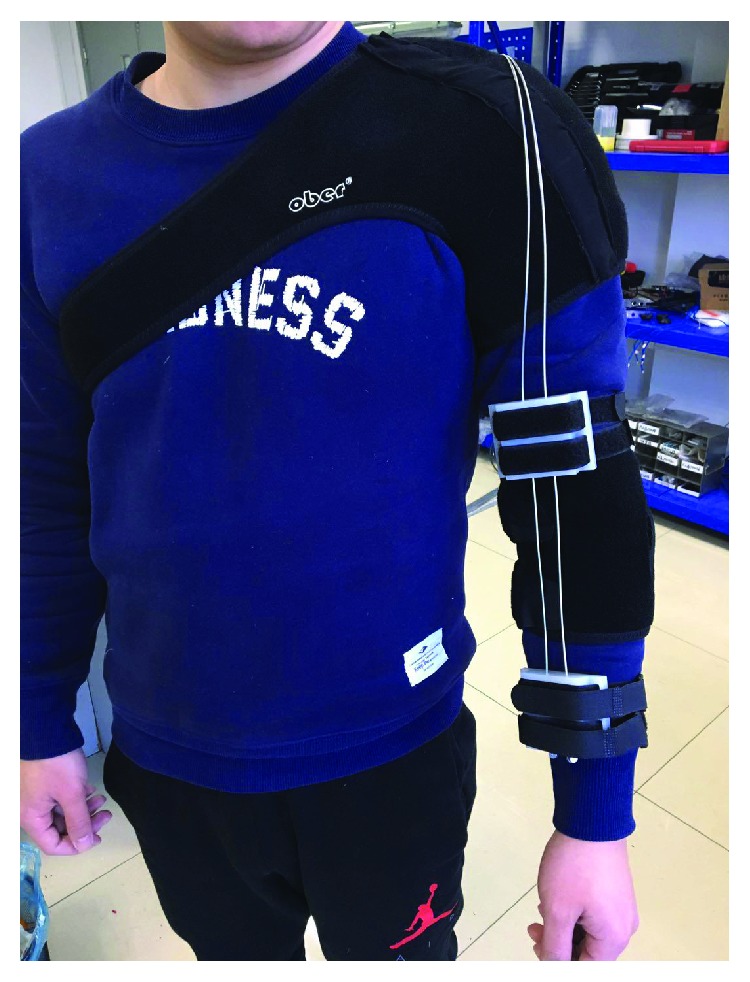
Subject with upper limb soft exoskeleton.

The purpose of the experiment is to demonstrate that the proposed upper extremity soft exoskeleton structure optimization is effective. The human subject experiments were carried out on a 70 kg male subject. As is shown in Figure 8, a subject performs flexion and extension movements with upper limb soft exoskeleton. When wearing the upper limb soft exoskeleton, the subject can adjust the binding position through the Velcro to make sure fix binding belts in the right place accurately. The structural optimization described in the previous section is validated during these tests. During the experiment, the subject is instructed to perform multiple repetitive flexion movements with complete assistance provided by the soft exoskeleton. The center of the forearm is defined as the middle position. The position which extends three meters from the middle position to the wrist is defined as the position away from the elbow. The position which extends in the opposite direction is defined as the position close to the elbow. Two experiments are carried out separately.

1. Based on the fixed position of the connecting point between the soft exoskeleton and the arm, multiple repetitive flexion movements of the elbow are performed with complete assistance delivered by the exoskeleton when the number of the Bowden cables is changed.
2. On the basis of a fixed number of Bowden cable, multiple flexion movements of the elbow are performed with complete assistance offered by the exoskeleton when the connection position between the exoskeleton and the arm is changed.

A film pressure sensor mounted between the body and the soft exoskeleton detects the pressure on the surface of the human body *F*. The flex sensor fixed on the elbow detects the motion for elbow *ϕ*_e_. Pressure *F* and angle *ϕ*_e_ are fitted through MATLAB to get the curves of pressure and motion angle.

### 4.2. Experimental Results

**Figure 9:**
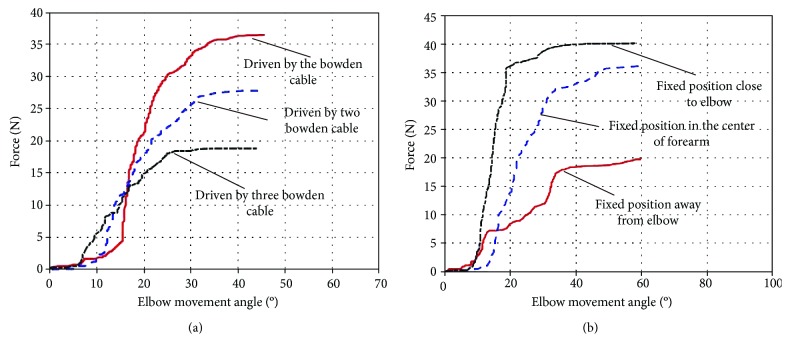
(a) Pressure comparison on different numbers of Bowden cable with exoskeleton's assistance. (b) Pressure comparison on different fixed positions with exoskeleton's assistance.

Results of the first experiment comparing the different numbers of the Bowden cables are depicted in Figure 9(a), showing that the pressure on the human body surface is reduced in pace with the increase of number of Bowden cables. As shown in Figure 9(a), man-machine interaction forces are almost same until flexion angle of elbow is 18 degrees. Afterwards, the man-machine interaction force is reduced with the increase of number of Bowden cables. The experimental results verify the conclusion with regard to the number of Bowden cables in the simulation experiment. The man-machine interaction force can be reduced when the number of force bearing points is increased in a certain range to spread the pressure over the wider range. Therefore, the safety and comfort of the exoskeleton system can be improved by increasing the number of Bowden cables. The results of the second experiment comparing the different connection positions between Bowden cable and the webbing strap are depicted in Figure 9(b), showing that the man-machine interaction force is reduced when force bearing point is away from the elbow. As shown in Figure 9(b), man-machine interaction forces are almost the same until flexion angle of elbow is 10 degrees. Afterwards, the man-machine interaction force is decreased when the force bearing point is away from the center of elbow. The experimental results verify the conclusion about the fixed position in the simulation experiment. Man-machine interaction force can be reduced when then connection point between Bowden cable and webbing strap is away from the center of elbow in a certain range.

## 5. Conclusions and Future Work

We propose the design of the mechanical structure and actuator for upper limb soft exoskeleton based on Bowden cable. Methods of structural optimization are proposed to reduce the man-machine interaction force. Human arm model with upper limb soft exoskeleton is established in ADAMS. Through simulation experiments, the influence of the number of force bearing points and the fixed position of force bearing point on man-machine interaction force in a certain range is summarized. A pilot evaluation demonstrates conclusions of simulation experiments. The subject with upper limb soft exoskeleton performs flexion movements of the elbow with assistance provided by soft exoskeleton under the condition of different numbers of Bowden cables and different connection points between Bowden cable and webbing strap. The following conclusions are obtained.

1. The man-machine interaction force can be reduced when the number of Bowden cables is increased to distribute pressure in a wider range within measure.
2. The man-machine interaction force can be reduced when the connection position is changed to make force bearing point away from the center of the elbow.

Based on the optimized structure proposed in the paper, the upper limb soft exoskeleton is suitable for the recovery training of stroke patients. This optimized structure can reduce the man-machine interaction force by 10%–15% and avoid the damage to patients.

The soft exoskeleton is extremely light and hardly causes scratch. This soft exoskeleton also does not limit motion freedom basically and allows the wearer to move through their full range of motion.

There are many areas for future improvement of the exoskeleton system. At present, a simple actuation is built to facilitate the structure optimization. In the future, the motor and gearbox need to be systematically selected and the structure of actuation should be further optimized to improve the transmission and reduce the friction of the Bowden cable transmission system. Friction is temporarily neglected in the design of the control system. In the actual situation, the control system needs to be improved and friction factor needs to be considered. In the future, patients with apoplectic hemiplegia will participate in the experiment to verify the accuracy of the hierarchical control system and evaluate the effect of the exoskeleton.

## Data Availability

All the data supporting the results were shown in the paper and can be available from the corresponding author.
